# P091: Timely administering prophylactic antibiotics

**DOI:** 10.1186/2047-2994-2-S1-P91

**Published:** 2013-06-20

**Authors:** Y Yau, I Wong

**Affiliations:** 1 Nursing ICNT, Hong Kong, Hong Kong; 2 Nursing CND, DKCH, Hong Kong, Hong Kong

## Introduction

It is well documented that prophylactic antibiotic could reduce surgical site infection significantly providing that it is given timely with right drug and right dose. However, administering prophylactic antibiotic in a timely manner is not easy. Several factors such as low awareness of importance of timing on prophylactic antibiotic given, the workflow, ease of administration and perception of individual responsibility toward the administration could all contribute to the failure of given timely antibiotic to reduce surgical infection.

## Objectives

To investigate how these problems were tackled in an elective surgical hospital in order to achieve timely administering surgical prophylactic antibiotic.

## Methods

During the period 2009 to 2012, all anesthetic records of orthopedic operation performed at the Duchess of Kent Children’s Hospital at Sandy Bay were reviewed. Data on time, administrator of prophylactic antibiotic, logistic of antibiotics being issued, methods of safety guide for administration and infection control rate were collected.

## Results

- Total 6061 cases were reviewed.

- 99.9% of the prophylactic antibiotics were given within 15-45 minutes interval before operation.

- 100% cases went through the safety check list by nurse.

- Anesthesiologist administered all the antibiotics while surgeon prescribed all prophylactic antibiotics.

- All prophylactic antibiotics were given in operating theater except Vancomycin.

- However, 99% antibiotics went through their regular route to OT.

- Infection rate was 0.13% over these 4 years.

## Conclusion

Clearly defined roles and responsibility in the process of prophylactic antibiotic administration were essential. Safety check list helped to enforce the guidelines. Monitoring the outcome alerted stakeholders to take necessary action. Successful transfer evidence-based guidelines into daily practice required multiple interventions and support from top management to frontline staff were vital.

## Disclosure of interest

None declared.

